# Differential Processing for Actively Ignored Pictures and Words

**DOI:** 10.1371/journal.pone.0170520

**Published:** 2017-01-25

**Authors:** Maegen Walker, Margeaux Ciraolo, Andrew Dewald, Scott Sinnett

**Affiliations:** Department of Psychology, University of Hawaiʻi at Mānoa, Honolulu, Hawaiʻi, United States of America; Plymouth University, UNITED KINGDOM

## Abstract

Previous work suggests that, when attended, pictures may be processed more readily than words. The current study extends this research to assess potential differences in processing between these stimulus types when they are actively ignored. In a dual-task paradigm, facilitated recognition for previously ignored words was found provided that they appeared frequently with an attended target. When adapting the same paradigm here, previously unattended pictures were recognized at high rates regardless of how they were paired with items during the primary task, whereas unattended words were later recognized at higher rates only if they had previously been aligned with primary task targets. Implicit learning effects obtained by aligning unattended items with attended task-targets may apply only to conceptually abstract stimulus types, such as words. Pictures, on the other hand, may maintain direct access to semantic information, and are therefore processed more readily than words, even when being actively ignored.

## Introduction

The successful completion of a complex activity, such as driving a car, often depends on the ability to ignore task-irrelevant information. While there is much debate over the extent to which ignored information is processed, there is little doubt that ignored information can be processed and potentially influence behavior [1–7]. The degree to which ignored information is processed is dependent on a number of factors. Critically, certain conditions appear to lead to facilitated processing for ignored information such that it may be recognized more readily at a later time. For example, frequently presenting ignored information in temporal synchrony with items that are attended leads to perceptual enhancements for the unattended items [2,7–11].

Empirical evidence in support of facilitated processing for ignored information can be seen in research conducted by Watanabe and colleagues ([7] see also [2]). In these examples, researchers paired irrelevant, subthreshold motion directions (i.e., dynamic dot displays that moved in a coherent motion direction below the threshold of conscious perception) with a target during an attended letter identification task. Participants were asked to hit a button when they saw a target grey letter that was displayed amongst a series of distractor black letters in a rapid serial visual presentation (RSVP) stream. Critically, when the target grey letters appeared, 5% of the dots in the irrelevant background display moved in a coherent motion direction (e.g., 0°) and when the distractor black letters were shown, the dots in the display moved at random [7] or in a different coherent motion direction (e.g., 90°) [2]. After this task was completed, a surprise recognition test measured participants’ ability to detect those same motion directions at suprathreshold levels. The findings suggested that subthreshold motion directions that had previously been paired with the target grey letters were perceived better than the motion directions that had been paired with the distractor black letters (note, all motion directions were subthreshold during the initial presentation, regardless of their alignment condition). This was taken as evidence that task-specific adaptive learning [7] may proceed for information that was presented below thresholds for explicit awareness, provided that it was frequently and simultaneously paired with a target during an attention-demanding task.

Dewald and colleagues [10] extended this finding to more complex semantic stimuli by using written words and pictures. Here participants were presented with a RSVP stream of line drawn pictures with irrelevant distractor words superimposed on top (i.e., temporally and spatially paired). In this task, the pictures were the attended items, while the words were the ignored stimuli. Similar to the task described above [2,7], participants were asked to hit a button any time they saw a picture immediately repeat in the stream, which served as the targets in this task, while ignoring the superimposed words (see methods for a full description of the task). Afterward, participants were given a surprise recognition test for the ignored words. Consistent with Watanabe et al. [2,7], during the surprise recognition test, participants recognized the words that had been paired with target picture repetitions more often than other previously ignored words, despite the fact that all word types had been presented in equal frequency. This finding demonstrates that adaptive learning, observed with simple visual stimuli by Watanabe et al. ([7] see also [2]), extends to more complex semantic visual stimuli (i.e., words). The robustness of these findings has also been observed in extensions involving auditory and cross-modal presentations [8,9,11], however, these studies have focused exclusively on examining later recognition rates for unattended *words*.

The extent to which these findings will generalize to other types of complex semantic stimuli, such as pictures, remains unknown, thereby driving the necessity to ascertain the differences between processing words and pictures when these items are attended to as well as actively ignored. This is particularly relevant when considering the amount of information that is presented to an individual on a daily basis in both pictorial and written formats (e.g., street and safety signs). Though a distinct overlap in neurological pathways has been observed [12,13], previous work suggests that, under conditions of directed attention, words may be processed differently from non-word semantic stimuli such as pictures [14–17]. Indeed, Amit et al. [14] hypothesized that words are actually generic labels for categories of items and therefore maintain a high level of conceptual abstraction, whereas pictures are specific and explicit items in and of themselves, which leads to relatively low levels of conceptual abstraction. For example, the word *coffee cup* actually describes a class of items that can vary drastically from one another and comprehension of the meaning of the word is a semantic construction, whereas a picture of a coffee cup depicts a literal example of a particular shape and size without abstract distinction. Therefore, it is likely that processing pictures may be easier due to this stimulus type having a lower level of conceptual abstraction.

The notion of words having a higher level of abstraction, compared to pictures, has been robustly demonstrated in numerous studies exploring the conditions that lead to each stimulus type (pictures or words) being processed more efficiently [14,15,17]. Performance for pictures is better (i.e., faster reaction times) when the items must be categorized, while faster reaction times are observed for written words when items must be named [15–17]. These studies arguably demonstrate that pictures capture more direct access to semantic information because object recognition is typically considered to involve the retrieval of visual and abstract semantic information, which occurs prior to the retrieval of lexical or name representations [18,19]. Words may take longer to categorize because their higher level of conceptual abstraction requires that they must first be decoded before they can be semantically evaluated (i.e., they must be read first and then associated with a meaning). On the other hand, naming may be faster for words because accessing semantic information is not necessary for reading a word, while it can be argued that this must still occur in order to name a picture [15,20].

Previous research has focused on how pictures and words are processed differently when they are attended, however, it is not well understood how these types of stimuli may be processed differently when they are actively ignored. To the best of our knowledge, few studies have focused on making this determination using cross domain approaches to explore differences between word and picture processing, with the exception of some related work by Tipper and colleagues [21,22]. These researchers demonstrated that semantic information is indeed accessed for both pictures and words when these items were actively ignored. Results from their studies showed that ignored words and pictures can elicit negative priming across domains: meaning that ignored words can lead to negative priming for pictures of the same category and vice versa. This finding was supported by later research [23] that demonstrated activation of the anterior temporal cortex, an area widely thought to be involved in semantic processing, in a similar negative priming paradigm. Though it should be noted that this work involved the investigation of pictures only and did not address neural correlates associated with word processing in negative priming paradigms (see [24,25] for examples of N400 processing for ignored words using even related potentials (ERP)).

It is possible that the readiness with which pictures have access to semantic information may have an impact on how this type of information is processed when it is actively ignored. Indeed, there is evidence to suggest that when pictures are actively ignored they may be identified beyond their physical features resulting in activation of phonological processing regions such as Wernicke’s area and the posterior inferior frontal gyrus, implying that the name of the item may be retrieved by linguistic areas [26]. However, our understanding of picture processing is limited in paradigms utilizing both words and pictures to explore the fate of ignored information. Previous approaches have focused exclusively on recognition performance of ignored words, using the pictures only as the attended stimuli in the primary task [1,4,10,11,25,27]. Other investigations have examined the effects of pairing irrelevant images with attended tasks [28,29], although the distractor images were explicitly attended along with the primary identification task. Therefore, this body of research does not examine the extent to which actively ignored items may be processed.

The current study aims to address these gaps in the literature with the focus being twofold: 1) to determine the extent to which previously observed adaptive learning for ignored words may extend to other forms of complex semantic stimuli (i.e., pictures), and 2) to contribute to a body of knowledge exploring how words and pictures may be processed differently under conditions in which these items are actively ignored. This is accomplished by employing the same paradigm that was used by Dewald et al. [10], but with a modification that entails a performance measure for unattended images rather than unattended words, during a surprise recognition test. The use of this paradigm allows for specific predictions to be made. First, because pictures appear to have more direct access to semantic representations, and this appears to result in pictures being processed more efficiently than words, it is reasonable to predict that, overall, unattended pictures may be recognized more often than unattended words. Furthermore, given the number of studies demonstrating that the frequent pairing of an unattended item with a target item in an attended task leads to facilitated processing of the unattended item, we predict that items paired with targets (pictures or words) during the primary task will be recognized significantly more often than other ignored items during the surprise recognition test.

## Methods

### Participants

Thirty-four participants (22 female, mean age of 19.9 years) were recruited for the “unattended pictures” condition. Data for two participants were not analyzed as they failed to respond during the repetition detection task. Fifty-three participants (33 female, mean age 20.8 years) were recruited for the “unattended words” condition. Data for two participants were not analyzed as they failed to respond during the repetition detection task. Of the remaining 51 participants, a subset of 32 (18 female, mean age of 20.6 years) were randomly selected and used for data analysis. Therefore the reported analyses contain a total of 64 participants (32 for each condition). This was done to ensure data met criteria for statistical diagnostics. All participants were recruited from the University of Hawaiʻi at Mānoa in exchange for course credit. Participants were naïve to the experiment, provided oral informed consent, and had normal, or corrected to normal, vision and hearing. Oral consent was obtained in order to ensure that participation was anonymous and in line with University of Hawaiʻi at Mānoa IRB regulations that no identifying information be collected from participants. Verbal consent was recorded in the aggregated participant log attached to a randomly generated identification number assigned to each participant. This research was approved by the University of Hawaiʻi at Mānoa Institutional Review Board, approval CHS #21455.

### Stimuli

A total of 50 pictures (on average 5 to 10 cm) were selected from the Snodgrass and Vanderwart [30] picture database. These pictures were randomly rotated +/-30 degrees from their original orientation to ensure that the primary task was sufficiently demanding in each version of the experiment [4]. Each picture was superimposed with one of two hundred high frequency English words selected from the MRC psycholinguistic database [31]. The words had an average length of 5 letters with a range of 4–6 and average frequency of 361 per million with a range of 135–782. Care was taken to ensure that picture-word combinations did not have any semantic relationship. The words were superimposed over the rotated pictures in bold, capitalized letters and presented in Arial font (24 points). For the primary task during the exposure stage, a stream of 960 combined picture-word items (height and width not exceeding 10 cm) was created and presented in a RSVP stream.

#### Unattended Pictures Condition

For the condition in which participants attended to words during the primary task and were tested on the ignored pictures during the surprise recognition test, 50 words and eight pictures were selected. Repeated words in the RSVP stream acted as the task-relevant targets in the primary task. The RSVP stream was broken into eight blocks of 120 trials. The presentation was pseudorandomized so that in each block an immediate word repetition occurred an average of one out of every eight trials, creating a mean of 15 task-relevant targets (word repetitions) per block. This resulted in a total of 120 trials of exposure to a task-relevant target (word repetition) and a simultaneously presented task-irrelevant picture.

Of the eight pictures that were selected and superimposed with words in the 960 trial RSVP stream, one was randomly selected to appear in temporal alignment (i.e., target-aligned or TA) with every task-relevant target (i.e., immediate word repetition). All other pictures appeared superimposed with words that did not immediately repeat (i.e., non-aligned or NA). In other words, a single picture was selected and always paired with the presentation of an immediate target word repetition, however all pictures were presented an equal number of times during the exposure stage. Eight iterations of this condition were created for which each of the eight pictures acted as the image that was aligned with the target word repetitions. To control for any possible differences that may have existed with regard to individual picture saliency, the presentation was randomized between participants. This was done to replicate the dependent measure and parallel the quantity of items and exposure to irrelevant stimuli employed by Dewald and colleagues ([10,8] see also [7]).

#### Unattended Words Condition

The exact same experimental construction was employed for the condition in which participants attended to the picture stream and were tested on ignored words. Here, 50 pictures and eight words were selected. Immediate picture repetitions served as the task-relevant target while a single word was selected and always paired with the presentation of an immediate target picture repetition (TA). As before, all other words appeared superimposed with non-repeating pictures (NA) in the RSVP stream, with all words presented an equal number of times. Again, to control for possible differences in individual word saliency, eight versions were created in which each word served as the TA word.

#### Surprise Recognition Test

The surprise recognition tests consisted of a total of either sixteen pictures or words (depending on the condition of the exposure stage). For those in the “unattended pictures” condition, participants were tested on the previously ignored pictures. For those in the “unattended words” condition, participants were tested on the previously ignored words. For each version of the surprise recognition test, eight items (pictures or words) came from the previously viewed visual stream (the seven NA items and one TA item), while the other eight consisted of never before seen foil items. The foil items were never used in the exposure stage of the experiment, but were taken from the same picture or word databases.

The recognition tests were randomized and presented by DMDX software [32]. The pictures were presented in the same manner as during the exposure stage, without words superimposed on top, and remained on the screen until a response was made. The words were also presented in the same manner as during the exposure stage, without pictures superimposed, in bold Arial font at 24 point and remained on the screen until a response was made.

### Procedure

**Fig 1:**
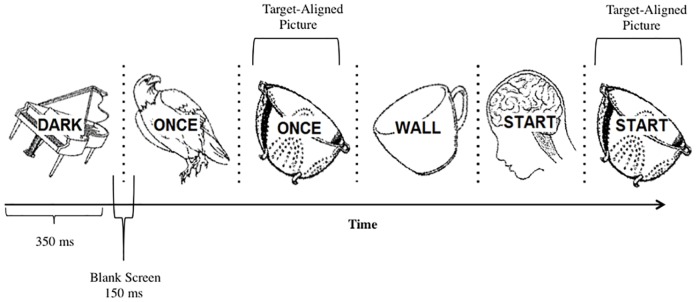
Schematic representation of the primary task in the unattended pictures condition. Repeated words serve as the target in the identification task (e.g., “once” and “start”) while superimposed images are the ignored items. Pictures appearing with immediate word repetitions are the TA items (e.g., the colander); all other pictures are NA items. Notice that the TA item is always the same. However, all ignored items are presented an equal number of times during the entirety of the primary task.

Depending on the assigned condition (i.e., unattended pictures or unattended words) participants were asked to attend to one aspect of the visual stream (words or pictures), while ignoring the other. They were instructed to respond when they saw a target item (words or pictures) immediately repeat in the RSVP stream by clicking the left mouse button with their preferred hand. Each item in the picture-word presentation was presented for 350 ms with a 150 ms inter-stimulus interval (ISI; blank screen) between each item for a stimulus onset asynchrony (SOA) of 500 ms (see Fig 1). Before the first experimental block, a training block of eight trials was given and repeated until participants were familiar and comfortable with the task. Immediately after the primary task, the surprise recognition test for the unattended items was administered. Participants were instructed to press the “B” key if they had seen the picture (or word) during the primary task or, instead, the “V” key if they had not seen the picture (or word) before. Response keys were counterbalanced across participants.

## Results

### Immediate Repetition Accuracy

Overall performance accuracy of immediate target repetition identification revealed that participants were successful at detecting target repetitions (pictures or words) in the primary task, with proportion of hits (*M* = 0.57, *SE* = 0.02) significantly better than chance, (*t*(63) = 31.5, *p* < 0.001). There was no significant difference in the accuracy score (total hits minus false alarms) between the two groups (word repetitions: *M* = 66, *SE* = 2.76 vs. picture repetitions: *M* = 63.2, *SE* = 2.5, *t*(62) = 0.743, *p* = 0.460). Overall, false alarms (FAs) were very low for all participants (M = 0.01, SE = 0.004). Participants attending to word repetitions had significantly more FAs (M = 0.006, SE = 0.003) than participants attending to picture repetitions (M = 0.003, SE = 0.004, *t*(62) = 2.96, *p* < 0.01). The higher rate of FAs among those attending to word repetitions may be due to greater similarities between visual features among individual words compared to pictures, although given the very low false alarm rates it is difficult to draw a precise conclusion.

### Overall Recognition Performance

Overall, participants were able to successfully distinguish between old items (i.e. previously ignored words or pictures from the primary task) and the novel foil items in the surprise recognition test with a significantly higher proportion of hits (i.e., correct identification of old items) than FAs (i.e., incorrectly identifying a foil item as having been present during the primary task), (Hits: M = 0.76, SE = 0.02, FAs: M = 0.14, SE = 0.03, *t*(63) = 18.28, *p* < 0.001). There were also significantly fewer FAs than predicted by chance (*t*(67) = 14.15, *p* < 0.001).

**Fig 2:**
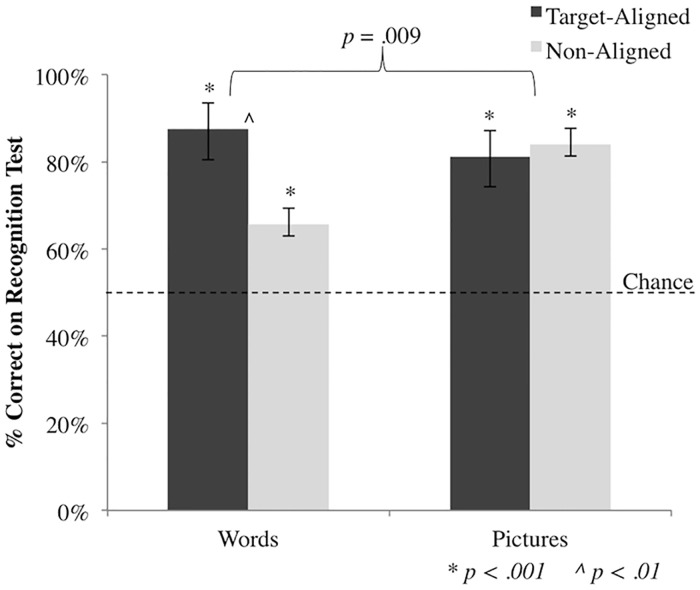
Performance accuracy on the surprise recognition test. Recognition rates and standard error for TA items (dark grey bar) and NA items (light grey bar). “Words” and “Pictures” indicate the stimulus type in the recognition test. Asterisks (*) indicate a significant difference from chance; caret (^) indicates a significant difference between items within a group.

To assess the influence of target-alignment on stimulus recognition in the surprise recognition test, a two-factor ANOVA was conducted on recognition performance for the surprise recognition test, with focus of attention (unattended pictures or unattended words) as the between subjects factor and alignment (TA or NA) as the within subjects factor and accuracy for TA and NA items as the dependent variable. There was a main effect for target-alignment indicating that, overall, TA items (*M* = 0.84, *SE* = 0.05) were recognized significantly more often than NA items (*M* = 0.75, *SE* = 0.03, *F*(1,62) = 4.40, *p* = 0.04, despite all items having been presented an equal number of times. There was no main effect for group type (unattended pictures vs. unattended words) indicating that despite the numerical trend, unattended pictures (*M* = 0.83, *SE* = 0.04) were not recognized significantly more often than unattended words (*M* = 0.77, *SE* = 0.04, *F*(1,62) = 1.14, *p* = 0.290). However, an interaction was observed (*F*(1,62) = 7.20, *p* = 0.009), suggesting that ignored items were recognized differently according to group type (Fig 2). This interaction was further explored in planned comparisons for each condition (unattended pictures or words).

### Unattended Pictures

Overall, participants were able to recognize the previously unattended pictures statistically better than chance (*M* = 0.84, *SE* = 0.03, *t*(31) = 12.11, *p* < 0.001). Recognition for the TA pictures (*M* = 0.81, *SE* = 0.07, *t*(31) = 4.46, *p* < 0.001) and the NA pictures (*M* = 0.84, *SE* = 0.03, *t*(31) = 12.92, *p* < 0.001) was also better than chance, but not different from each other (*t*(31) = 0.419, *p* < 0.678) (Fig 2).

### Unattended Words

Overall, participants were able to recognize the previously unattended words statistically better than chance (*M* = 0.68, *SE* = 0.04, *t*(31) = 5.24, *p* < 0.001). Recognition for the TA words (*M* = 0.86, *SE* = 0.06, *t*(31) = 6.31, *p* < 0.001) and the NA words (*M* = 0.66, *SE* = 0.04, *t*(31) = 4.13, *p* < 0.001) was each better than chance. Furthermore, and driving the interaction, TA words were recognized more accurately than NA words (*t*(31) = 3.34, *p* = 0.002) (Fig 2).

### Analysis by Target-Alignment

Comparing performance on the surprise recognition test for TA and NA items between each group type revealed that there was no significant difference in recognition rates for TA items between the unattended words (*M* = 0.86, *SE* = 0.06) and unattended pictures (*M* = 0.81, *SE* = 0.07, *t*(62) = 0.68, *p* = 0.499). However, NA words (*M* = 0.66, *SE* = 0.04) were recognized significantly less often than NA pictures (*M* = 0.84, *SE* = 0.03, *t*(62) = 3.97, *p* < 0.001) (Fig 2).

## Discussion

Earlier research suggests that the extent to which unattended words are processed, and recognized later, may be facilitated when they are frequently and simultaneously paired with targets during a previously presented attention-demanding task [8–11]. However, the degree to which these findings may generalize to other forms of semantic stimuli, such as pictures, is yet unknown. A sizeable body of research has investigated how pictures and words may be processed differently under conditions in which these items are explicitly attended [12–17], suggesting that attended pictures are processed more readily than attended words because they may maintain more direct access to semantic representations. Nevertheless, there are few studies that have examined how these types of stimuli may be processed differently under conditions in which these items are actively ignored (but see [21,22] for examples of negative priming). Therefore, the current study aimed to determine the extent to which previously observed facilitatory effects for ignored words will extend to ignored pictures, as well as contribute to current understanding of how these items may be processed differently under conditions in which attention is explicitly directed elsewhere.

Given that pictures appear to be processed more efficiently than words when attention is directed toward these items, we anticipated that, overall, recognition rates for unattended pictures would be higher than unattended words. While we failed to observe a main effect for this comparison, the significant interaction indicated a difference in the recognition pattern between the two groups. Participants in the *unattended pictures* condition did appear to exhibit higher recognition rates compared to those in the *unattended words* condition, although the higher performance was limited to recognition rates for the NA items only (ignore pictures: TA = 81% vs. NA = 84%; ignore words: TA = 86% vs. NA = 66%). This finding potentially suggests that participants in the *unattended pictures* condition experienced facilitation for all item types (TA and NA) while those in the *unattended words* condition demonstrated facilitated recognition rates for TA items compared to NA items. Based on findings from previous research [7,8,10,11] we also expected that TA items (pictures and words) would be recognized more often than NA items. A main effect for target-alignment was observed, suggesting that TA items were, indeed, recognized more often than NA items. This finding is in concert with previous research examining the extent to which explicitly ignored *words* are processed [8–11], however, participants were able to identify previously ignored pictures during the surprise recognition test very well regardless of whether they were aligned with a target or not.

Earlier studies demonstrated that both pictures and words are processed at the semantic level under conditions in which these items are actively ignored [21,22,33]. However, written words still require an additional step (i.e., they must still be read) to access semantic information. Therefore, it is possible that this additional step in processing leaves words more susceptible to decay once the information has been entered into working memory. This decay may be attenuated when the unattended words frequently appear in temporal synchronicity with attended task targets. That is, due to the fact that the TA words always appear with an attended task target, these items may undergo extended processing compared to the other NA words despite all stimuli being presented an equal number of times.

It is unlikely that the enhanced recognition for TA words may be attributed to heightened arousal resulting from target identification, as this was not observed when comparing TA and NA pictures. That is, if heightened arousal were solely responsible for the enhanced recognition of previously unattended stimuli, we would have expected to see a similar trend between pictures and words. While it is possible that the overall high accuracy score for unattended pictures may have masked arousal related increases in response accuracy, previous research conducted by Dewald and Sinnett [34] suggests that this is interpretation is still unlikely. Their study compared recognition rates for TA and NA words when participants either ignored or attended the word stream. They found that when attention was directed away from the words (i.e., participants monitored a picture stream for targets) then TA words were recognized significantly more often than NA words (as seen in the current study). However, when participants attended the word stream, recognition rates between target words and non-target words (analogous to TA and NA words when they are ignored) did not differ. Thus, if heightened arousal, resulting from target detection, were indeed responsible for the higher recognition rates, we would expect to see the same trend in results when items are attended (i.e., higher recognition rates for target words compared to non-target words). Therefore, the heightened recognition rates for unattended words is likely due to associations formed between the attended and ignored stimuli. Indeed, Seitz and Watanabe [7] argue that when irrelevant information, such as motion direction, is paired with a task target, it may become bound by internal reward signals generated from successful target identification, thereby linking this particular stimulus with task performance, leading to higher recognition rates later. This research is supported by more recent work by Seitz, Kim, and Watanabe [35] suggesting that external rewards may enhance learning of irrelevant visual stimuli even when a behavioral response is not made. However, this interpretation may be incomplete as the previously observed facilitation for TA items may be stimulus dependent, applying only to more abstract items such as words.

While Seitz and Watanabe’s [7] findings extend to unattended words, this does not seem to be the case for unattended pictures. Again, recognition for pictures was not modulated by whether these items were paired with a target or not and overall accuracy was quite high. It is unlikely that the higher recognition rates for pictures when compared to words can be attributed to differences in primary task difficulty, as participants were equally successful at detecting targets during the primary task regardless of condition. One may argue that identifying word repetitions may have been marginally more difficult than identifying picture repetitions, as participants in the attend to words condition had significantly more false alarms than participants in the attend to pictures condition (*p* < 0.01). However, this would not account for overall higher accuracy scores when identifying unattended pictures during the surprise recognition test. If identifying word repetitions was more difficult, then we would expect to see lower accuracy scores during the surprise recognition test for unattended pictures, but this was not the case. Therefore, higher recognition rates could be attributed to notion that pictures are more conceptually concrete [14]. In turn, this would suggest that pictures reach a deeper level of processing more expediently during the primary task and the additional amount of processing that may occur by virtue of being paired with a task target may not have the same effect. That is, the reduced effort with which pictures are processed results in performance enhancements for this stimulus type that are near ceiling. Therefore, it is difficult to observe any additional facilitation that may occur for TA pictures. Alternatively, pictures may be recognized at higher rates than words during the recognition test because they may be more perceptually rich. However, this does not conflict with our interpretation that they may also be more conceptually concrete and easier to process. If pictures are indeed more perceptually rich than words, this would only serve to facilitate semantic processing for those items, which would contribute to those items being processed more readily.

Taken together, these results provide partial evidence for the facilitated processing for unattended information, dependent on the type of item being presented. While processing for unattended words seems to be facilitated when paired with an attended task target, processing for unattended pictures appears to be facilitated by virtue of this stimulus type. This suggests that unattended images may still have more direct access to semantic information, compared to words, under conditions in which these items are actively ignored. The more extensive processing for pictures also appears to negate the facilitatory recognition effects of temporal alignment of an unattended item with a target during a primary task, as has been typically observed in the literature with other stimuli types (i.e., motion directions and words, (see [7,10]). As a result of this undifferentiated perceptual enhancement for picture processing, these items may be stored more readily in long-term memory and recognized at overall higher rates later. It could be argued, as levels of processing theory [36] asserts, that information that is more “deeply” encoded is more likely to be retained in long-term memory. As previous research has demonstrated, words are processed to a greater extent when they must be categorized, while pictures are processed more deeply when they must be named—resulting in greater memory retention for each item depending on these conditions [15–17]. While no explicit instructions were given to participants regarding how they should identify each item type (i.e., by category or by name), it is possible that as each item was encountered, participants opted to name it as a strategy to complete the primary repetition detection task. This would facilitate “deeper” processing for the pictures and “shallower” processing for the words, which may attribute to the interaction that was observed here. However, as participants were instructed to actively ignore each item type of interest during the primary task, the point at which encoding would have taken place, it seems unlikely that this would have occurred while participants were identifying immediate target-repetitions because attention was directed away from the items they were ultimately tested on.

This study also replicated previous results from Dewald et al. [10], suggesting that when unattended *words* are frequently presented with a task target (TA), later recognition is enhanced compared to those words that did not appear with a task target (NA). This demonstrates the robust nature of adaptive learning effects for explicitly presented irrelevant words that had been temporally aligned with a previously presented task target. The findings reported here also extend the previous research on processing of irrelevant stimuli to include a condition in which participants were tested on explicitly ignored pictures. The fact that preferential facilitation for TA pictures was not observed suggests that learning effects obtained by aligning unattended items with attended task-targets may apply only to conceptually abstract stimulus types. The overall high recognition rates for unattended pictures, regardless of target-alignment, indicates that this stimulus type maintains direct access to semantic information, and is therefore processed to a greater extent, even under conditions of being actively ignored. While the current study lends support to the idea that there may exist differential processing for unattended pictures compared to words, as is seen when images and words are actively *attended* [14,15,17], more research is required to fully understand these observed differences. Future studies could examine the effect that cross-modal presentations of these stimuli types may have on later recognition rates.
